# Endogenous Avian Leukosis Virus in Combination with Serotype 2 Marek's Disease Virus Significantly Boosted the Incidence of Lymphoid Leukosis-Like Bursal Lymphomas in Susceptible Chickens

**DOI:** 10.1128/JVI.00861-19

**Published:** 2019-11-13

**Authors:** Jody K. Mays, Alexis Black-Pyrkosz, Tamer Mansour, Brian C. Schutte, Shuang Chang, Kunzhe Dong, Henry D. Hunt, Aly M. Fadly, Lei Zhang, Huanmin Zhang

**Affiliations:** a United States Department of Agriculture, Agricultural Research Service, Avian Disease and Oncology Laboratory, East Lansing, Michigan, USA; b Department of Microbiology and Molecular Genetics, Michigan State University, East Lansing, Michigan, USA; c Department of Clinical Pathology, School of Medicine, University of Mansoura, Mansoura, Egypt; d ORISE Fellow, USDA, Agricultural Research Service, Avian Disease and Oncology Laboratory, East Lansing, Michigan, USA; e U.S. Forest Service International Programs, Washington, DC, USA; f Institute of Special Wild Economic Animal and Plant Science, Chinese Academy of Agricultural Sciences, Changchun, Jilin, China; Ulm University Medical Center

**Keywords:** endogenous retrovirus, serotype 2 Marek’s disease virus, spontaneous tumors, genetic resistance, differentially expressed genes, signaling pathways

## Abstract

In 2010, sporadic cases of avian leukosis virus (ALV)-like bursal lymphoma, also known as spontaneous lymphoid leukosis (LL)-like tumors, were identified in two commercial broiler breeder flocks in the absence of exogenous ALV infection. Two individual ALV subgroup E (ALV-E) field strains, designated AF227 and AF229, were isolated from two different breeder farms. The role of these ALV-E field isolates in development of and the potential joint impact in conjunction with a Marek’s disease virus (MDV) vaccine (SB-1) were further characterized in chickens of an experimental line and commercial broiler breeders. The experimental line 0.TVB*S1, commonly known as the rapid feathering-susceptible (RFS) line, of chickens lacks all endogenous ALV and is fully susceptible to all subgroups of ALV, including ALV-E. Spontaneous LL-like tumors occurred following infection with AF227, AF229, and a reference ALV-E strain, RAV60, in RFS chickens. Vaccination with serotype 2 MDV, SB-1, in addition to AF227 or AF229 inoculation, significantly enhanced the spontaneous LL-like tumor incidence in the RFS chickens. The spontaneous LL-like tumor incidence jumped from 14% by AF227 alone to 42 to 43% by AF227 in combination with SB-1 in the RFS chickens under controlled conditions. RNA-sequencing analysis of the LL-like lymphomas and nonmalignant bursa tissues of the RFS line of birds identified hundreds of differentially expressed genes that are reportedly involved in key biological processes and pathways, including signaling and signal transduction pathways. The data from this study suggested that both ALV-E and MDV-2 play an important role in enhancement of the spontaneous LL-like tumors in susceptible chickens. The underlying mechanism may be complex and involved in many chicken genes and pathways, including signal transduction pathways and immune system processes, in addition to reported viral genes.

**IMPORTANCE** Lymphoid leukosis (LL)-like lymphoma is a low-incidence yet costly and poorly understood disease of domestic chickens. The observed unique characteristics of LL-like lymphomas are that the incidence of the disease is chicken line dependent; pathologically, it appeared to mimic avian leukosis but is free of exogenous ALV infection; inoculation of the nonpathogenic ALV-E or MDV-2 (SB-1) boosts the incidence of the disease; and inoculation of both the nonpathogenic ALV-E and SB-1 escalates it to much higher levels. This study was designed to test the impact of two new ALV-E isolates, recently derived from commercial broiler breeder flocks, in combination with the nonpathogenic SB-1 on LL-like lymphoma incidences in both an experimental egg layer line of chickens and a commercial broiler breeder line of chickens under a controlled condition. Data from this study provided an additional piece of experimental evidence on the potency of nonpathogenic ALV-E, MDV-2, and ALV-E plus MDV-2 in boosting the incidence of LL-like lymphomas in susceptible chickens. This study also generated the first piece of genomic evidence that suggests host transcriptomic variation plays an important role in modulating LL-like lymphoma formation.

## Abstract (precis)

Lymphoid leukosis (LL)-like lymphoma is a low-incidence yet costly and poorly understood disease of domestic chickens. The observed unique characteristics of LL-like lymphomas are that the incidence of the disease is chicken line dependent; pathologically, it appeared to mimic avian leukosis but is free of exogenous ALV infection; inoculation of the nonpathogenic ALV-E or MDV-2 (SB-1) boosts the incidence of the disease; and inoculation of both the nonpathogenic ALV-E and SB-1 escalates it to much higher levels. This study was designed to test the impact of two new ALV-E isolates, recently derived from commercial broiler breeder flocks, in combination with the nonpathogenic SB-1 on LL-like lymphoma incidences in both an experimental egg layer line of chickens and a commercial broiler breeder line of chickens under a controlled condition. Data from this study provided an additional piece of experimental evidence on the potency of nonpathogenic ALV-E, MDV-2, and ALV-E plus MDV-2 in boosting the incidence of LL-like lymphomas in susceptible chickens. This study also generated the first piece of genomic evidence that suggests host transcriptomic variation plays an important role in modulating LL-like lymphoma formation.

## INTRODUCTION

Lymphoid leukosis (LL) is a B-cell lymphoma of chickens taking place during 4 months of age and older (1, 2). The tumors typically involve liver, spleen, and bursa of Fabricius (3) and are usually composed of aggregates of lymphoblasts of B-cell origin with subsequent production of monoclonal IgM (4).

Commonly, LL is induced by transmissible strains of retroviruses, known as avian leukosis virus (ALV), in susceptible chickens. The specific strains of ALV are defined as exogenous ALVs, since they are transmitted as infectious virus particles. Exogenous ALVs propagate in most tissues and organs of the avian body but only persist in bursal lymphocytes, the target cells of neoplastic transformation (5). Field strains of exogenous ALVs do not harbor any oncogene but instead induce lymphoid leukosis by activation of the cellular *myc* oncogene (5). Only defective exogenous ALVs harbor oncogenes, such as v-*myc*, v-*src*, *v-myb*, etc., and have been shown to induce acute tumors in susceptible hosts (5).

Spontaneous ALV-like bursal lymphomas, also termed LL-like lymphomas, have been reported in chicken flocks in the absence of exogenous ALV (6, 7). These tumors are of bursal cell origin and are grossly and microscopically similar to exogenous ALV-induced LL but free of detectable ALV infection (7). Some genetic lines of chickens are more susceptible to the development of spontaneous ALV-like tumors, such as line 0 (8, 9) and the transgenic line *0.ALV6* (10), two of the chicken lines maintained by the USDA, Agriculture Research Service, Avian Disease and Oncology Laboratory (ADOL). The *0.ALV6* line, originally derived from line 0, carries a defective subgroup A avian leukosis provirus in its germ line and has been shown to develop spontaneous LL-like tumors similarly to line 0 (11).

There are seven subgroups, A, B, C, D, E, J, and K, of ALVs identified in chickens based upon the viral envelope glycoproteins (12–16). Unlike the other subgroups of exogenous ALVs, the subgroup E viruses are avian retrovirus-like elements that are transmitted genetically in a Mendelian fashion and are termed endogenous viruses (3). The domestic White Leghorn chicken genome carries at least 22 endogenous ALV proviral loci (*ev-1* through *ev-22*) (17–19). Many of the endogenous viruses are genetically defective and incapable of giving rise to infectious virions (20), whereas others may be expressed in an infectious form (21). In the infectious form, endogenous viruses are transmitted similarly to exogenous viruses, although there are chickens or lines of chickens that are genetically resistant to infection of endogenous viruses (3, 22, 23). Rous-associated virus type 0 (RAV-0), a subgroup E endogenous virus, has little or no oncogenic potential (24). However, RAV-60, a subgroup E recombinant of endogenous and exogenous viruses, is highly oncogenic, and infection of RAV-60 can lead to LL (25, 26). Endogenous ALVs also influence the response of birds to infection by exogenous ALV (9, 27–29).

Genetic resistance to avian leukosis occurs at two levels, cellular resistance to virus infection and resistance to tumor formation (30–33). Inheritance of cellular resistance to ALV infection is of a Mendelian type. Independent autosomal loci control the resistance to infection of subgroups ALV-A, -B, -C, and -J and are designated *tva* (tumor virus A subgroup), *tvb*, *tvc*, and *tvj*, respectively (22, 34–36). Receptors responsible for mediating the cellular resistance and infection for ALV subgroups D and E are also coded by the *tvb* locus of specific alleles (37).

The TVB receptor complex is coded by a series of three alleles (TVB*S1, TVB*S3, and TVB*R) of *tvb*. The TVB*S1 allele is dominant and encodes the receptor that mediates infection of ALV subgroups B, D, and E. The TVB*S3 allele is recessive to TVB*S1 but dominant to TVB*R and encodes a receptor that only mediates ALV-B and ALV-D infection, not ALV-E infection. The TVB*R allele encodes a defective (truncated) receptor incapable of facilitating infection by any of the three subgroup ALVs. Resistance to subgroup E ALV is more complex. In addition to the allelic forms of the *tvb* locus, it is believed that resistance to ALV-E also involves another locus, reportedly known as *i* (38). Subsequent studies suggested that the *i* locus is, in fact, a series of competent *ALV-E* inserts, also known as *ev* loci, that express high levels of the envelope glycoproteins of ALV-E, which, in turn, competently interfere with the binding processes between the ALV-E receptors and the subgroup ALV-E (23, 26, 39). Therefore, the susceptibility of both homozygous and heterozygous TVB*S1 chickens to subgroup ALV-E might be compromised by the replication-competent *ALV-E* inserts carried in those chickens’ genome (23).

Vaccination with Marek’s disease virus serotype 2 (MDV-2) has been shown to enhance the development of ALV and reticuloendotheliosis virus-induced bursal lymphomas (40–42) as well as spontaneous bursal lymphomas (11, 43). MDV-2 has been shown to elevate ALV gene expression and ALV replication (44). Furthermore, bursal cells coinfected with ALV and MDV-2 are more likely to be transformed (45).

In 2010, a commercial company observed the development of spontaneous LL-like tumors in broiler breeder flocks raised on more than one farm. The tumors were identified during postmortem examinations of hens between 35 and 45 weeks of age with no significant impact on reproductive performance and overall flock mortality. The incidence of birds with developed spontaneous tumors was sporadic and was found during routine postmortem exams. Lesions consistent with LL-like tumor were found in liver, spleen, kidney, and, less frequently, in other organs. Samples from various tissues received from the commercial broiler breeder chickens tested negative for known exogenous ALV of subgroups A, B, C, D, J, and K, as determined by virus isolation and PCR assays. However, two ALV-E field strains were isolated from two broiler breeder flocks raised on two different farms and were designated AF-227 and AF-229.

Since it was unclear if the two isolate-specific ALV-E field strains were responsible for the incidences of spontaneous LL-like bursa lymphomas observed in the commercial broiler breeder flocks, this study was designed to characterize the two field isolates by infecting birds sampled from a fully susceptible line of chickens, the line 0.TVB*S1, commonly known as the rapid feathering-susceptible (RFS) line, which lacks all endogenous ALV and is fully susceptible to all subgroups of ALV, including ALV-E (46). The affected commercial lines were no longer available at the time of this study; thus, a different line of commercial broiler chickens from the same commercial company was used in the challenge trials of the two field isolates in the presence and absence of vaccination with MDV-2. Complete and partial genome sequence analyses of the two field strains of ALV isolators were conducted for categorization and characterization. Total RNA samples were extracted from fresh LL-like bursa lymphoma tissues of the RFS chickens inoculated with AF227 followed by SB-1 vaccination, normal bursa tissues, and B cells isolated from normal spleen tissues of uninfected RFS chickens, which were subsequently subjected to RNA-sequencing (RNA-Seq) analysis to explore potential genomic variation that may reveal insights into the escalated incidences of spontaneous LL-like lymphomas.

## RESULTS

### The field isolates AF227 and AF229 are closely related to the ALV subgroup ALV-E.

**FIG 1:**
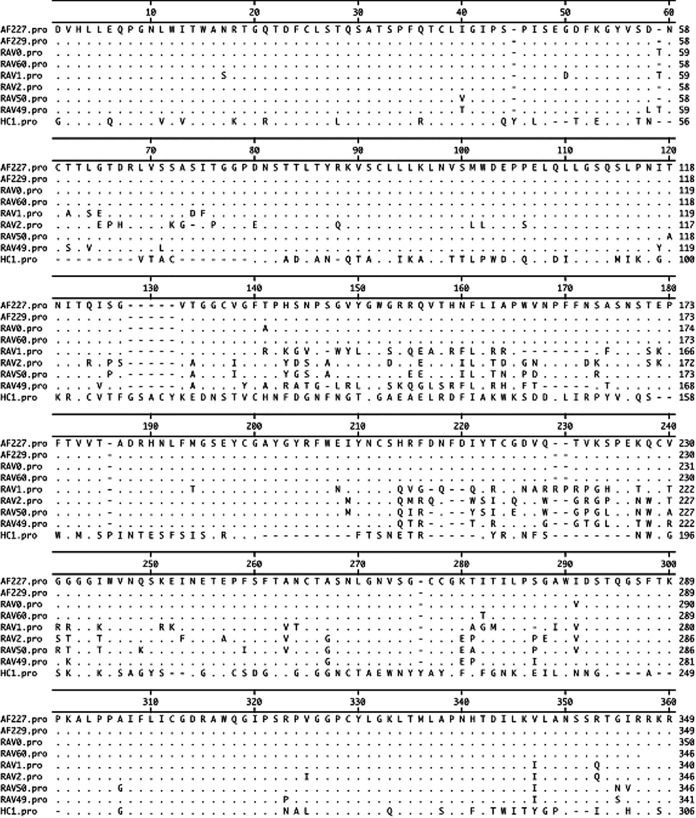
Comparison of gp85 amino acid sequences of avian leukosis virus (ALV) subgroups E (AF227, AF229, RAV0, and RAV60; accession numbers MF817820, MF817821, MF817822, and MF817823, respectively), A (RAV1, accession number MF926337), B (RAV2, accession number M14902.1), C (RAV49, accession number J02342.1), D (RAV50, accession number D10652.1), and J (HC-1, accession number AF247391.1). One-letter amino acid codes are listed. Amino acid residues identical to the majority of codes of AF227 are indicated by dots, dashes indicate amino acid residues missing due to deletions, and one-letter codes represent the differences between AF227 and a subgroup of ALV.

Two avian virus field isolates have been derived from commercial broiler breeder flocks. The genomes of the two field isolates, AF227 and AF229, along with the genomes of the prototype ALV-E strains, RAV-0 and RAV-60, and the prototype ALV-A strain, RAV-1, were sequenced. The complete genomic sequences have been deposited in GenBank. Accession numbers of MF817820 for AF227, MF817821 for AF229, MF817822 for RAV-0, MF817823 for a partial RAV-60 sequence, and MF926337 for RAV-1 were assigned. Sequence analyses of the complete AF227 and AF229 genomes were closely related to those of known endogenous ALV strains, which confirmed our expectation that these two new field isolates were subgroup E ALV, or ALV-E. The sequences of both AF227 and AF229 genomes were more than 99% homologous to the RAV-0 genome sequence. In contrast, the AF227 and AF229 genomes were only 89% homologous to the ALV-A (RAV-1) genome and 79% homologous to the ALV-J (HPRS-103) genome (47) sequences. Further comparison of the gp85 deduced amino acid sequences showed that AF227 and AF229 were 99% to 100% homologous to the sequence of the ALV subgroup E viruses, 81 to 85% homologous to those of ALV subgroup A to D viruses, and 42% homologous to that of ALV subgroup J viruses (Fig. 1). The amino acid sequences of the gp85 envelope protein of the endogenous viruses varied little but obviously differed from that of the exogenous viruses, consistent with the literature (48–52). Furthermore, there was only a 45% nucleotide sequence homology to exogenous long terminal repeats (LTRs), whereas the AF227 and AF229 LTRs showed 96% identity to those in ALV-E strain RAV-0. The endogenous ALV LTRs were 256 nucleotides long and were shorter than the exogenous RAV-1 LTRs, which were 347 nucleotides in length.

### Pathogenicity of the ALV-E field isolates AF227 and AF229.

The pathogenicity of the ALV-E field isolate AF227 observed in the fully susceptible RFS line of chickens is shown in Table 1. The RFS (C/0) chickens inoculated at 7 days of embryonation (DOE) were viremia tolerant for ALV-E, as evidenced by virus isolation at 4 and 32 weeks of age using RFS chicken embryo fibroblasts (CEFs). Cocultivation of buffy coat analysis showed that only the birds inoculated with MDV-2 in this trial were positive for MDV. No significant difference in the incidences of spontaneous LL-like lymphomas was observed following the inoculation of the RFS embryos at embryonation with either AF227 or serotype 2 vaccine (SB-1) alone on the day of hatch, which resulted in 14% and 17% of LL-like lymphomas in the RFS birds, respectively. However, inoculation of the RFS embryos at embryonation with both AF227 and SB-1 on the day of hatch significantly enhanced the incidence of LL-like lymphomas (*P* < 0.05), which resulted in 42% LL-like lymphomas in the RFS birds (Table 1).

**TABLE 1:** Incidences of LL-like lymphomas observed in RFS chickens inoculated with ALV-E AF227, MDV-2 vaccine (SB-1), or both

Inoculum	No. of chickens at risk	Virus isolation [no. of chickens positive/no. of chickens at risk (%)] at wk^*a*^:		No. of chickens with tumors/no. of chickens at risk^*b*^ (%)
		4	32	
PBS	24	0/10 (0)	0/10 (0)	2/24 (8)^a^
SB-1	24	0/10 (0)	0/10 (0)	4/24 (17)^a^
AF227	36	9/10 (90)	9/9 (100)	5/36 (14)^a^
AF227 + SB-1	36	10/10 (100)	10/10 (100)	15/36 (42)^b^

a Plasma samples were tested for endogenous ALV on CEFs of the ADOL line RFS (C/E).

b The inoculum groups not sharing a lowercase superscript letter differed in tumor incidences with statistical significance based upon chi-square analysis (*P* < 0.05).

The commercial broiler breeder flocks were typed by single-nucleotide polymorphism as TVB*S1/*S1 homozygous (data not shown) and should be fully susceptible to infection of ALV-E, provided that birds are free of *ev* gene expression, particularly *ev3*, *ev6*, and *ev21*, by the birds. Only 0 to 5% of the chickens at risk developed LL-like lymphoma following inoculation of SB-1 in combination with either AF229 or AF227. In contrast, 33 to 43% of RFS chickens developed LL-like lymphoma following the same challenges. All virus isolation results on the line RFS CEF were positive, except for those of the SB-1 and the phosphate buffered saline (PBS) inoculum groups of the RFS birds (Table 1). On the other hand, all virus isolation results on the line 0 CEF were negative for both the commercial broilers and the RFS birds at 4 and 32 weeks of age, which evidenced that there was exogenous ALV infection in neither the commercial boilers nor the RFS birds (Table 2).

**TABLE 2:** Incidences of LL-like lymphomas observed in commercial broiler breeders and RFS chickens inoculated with SB-1 MDV-2 vaccine, ALV-E isolate AF229, or a combination of SB-1 and AF227 or AF229

Inoculum	Chicken line	Virus isolation [no. of chickens positive/no. of chickens at risk (%)] at wk^*a*^:				No. of chickens with LL-like lymphoma/no. of chickens at risk^*b*^ (%)
		4		52		
		C/E	C/0	C/E	C/0	
PBS	Broiler	0/32 (0)	32/32 (100)	0/29 (0)	29/29 (100)	0/29 (0)^a^
AF229	Broiler	0/33 (0)	33/33 (100)	0/31 (0)	31/31 (100)	0/33 (0)^a^
SB-1	Broiler	0/35 (0)	35/35 (100)	0/31 (0)	31/31 (100)	0/35 (0)^a^
AF229 + SB-1	Broiler	0/35 (0)	35/35 (100)	0/30 (0)	30/30 (100)	0/34 (0)^a^
AF227 + SB-1	Broiler	0/27 (0)	27/27 (100)	0/14 (0)	14/14 (100)	1/19 (5)^a^
PBS	RFS	0/43 (0)	0/43 (0)	0/33 (0)	0/33 (0)	2/57 (3.5)^a^
AF229 + SB-1	RFS	0/33 (0)	33/33 (100)	0/19 (0)	19/19 (100)	11/33 (33)^b^
AF227 + SB-1	RFS	0/35 (0)	35/35 (100)	0/18 (0)	18/18 (100)	15/35 (43)^b^

a Plasma samples were tested for exogenous and endogenous ALVs on CEFs of ADOL lines 0 (C/E) and RFS (C/0).

b Tumor incidences between inoculum groups sharing no common lowercase superscript letter differed significantly based upon chi-square analysis (*P* < 0.05).

### Genomic analysis of AF227 by next-generation sequencing.

The entire nucleotide sequence of AF227 was recovered and assembled from RNA sequencing (RNA-Seq) data obtained from the LL-like bursal lymphomas of the AF227- and MDV-2-infected RFS chickens. The recovered AF227 nucleotide sequence was identical to that of the AF227 nucleotide sequence derived from cloned AF227 virus. In addition, the MDV-2 gene, R-LORF1, was detected with significantly high expression levels, while the SORF1 and SORF2 genes were expressed at a relatively low level in the LL-like lymphoma tissues.

### The malignant and nonmalignant bursa groups were more comparable on overall expression levels, in contrast to the splenic B-cell group.

**FIG 2:**
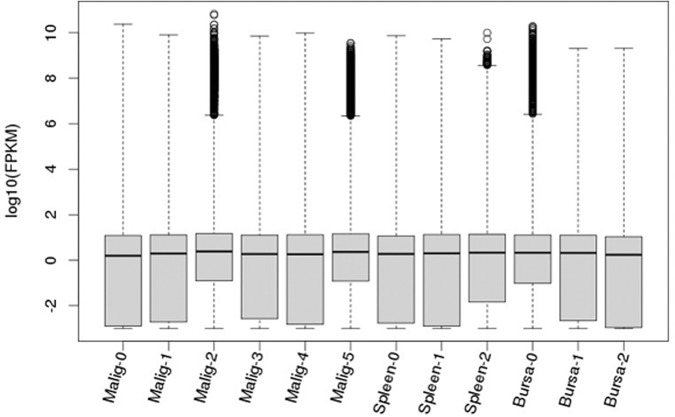
Box-and-whisker plots of gene expression profiles for each of the malignant samples (Malig-0 to Malig-5) and nonmalignant samples, including the control groups of bursa tissues (Bursa-0 to Bursa-2) and splenic B cells (Spleen-0 to Spleen-2), illustrating the RNA-Seq read distribution and variability of the RNA-Seq samples. FPKM, fragments per kilobase per million.

**FIG 3:**
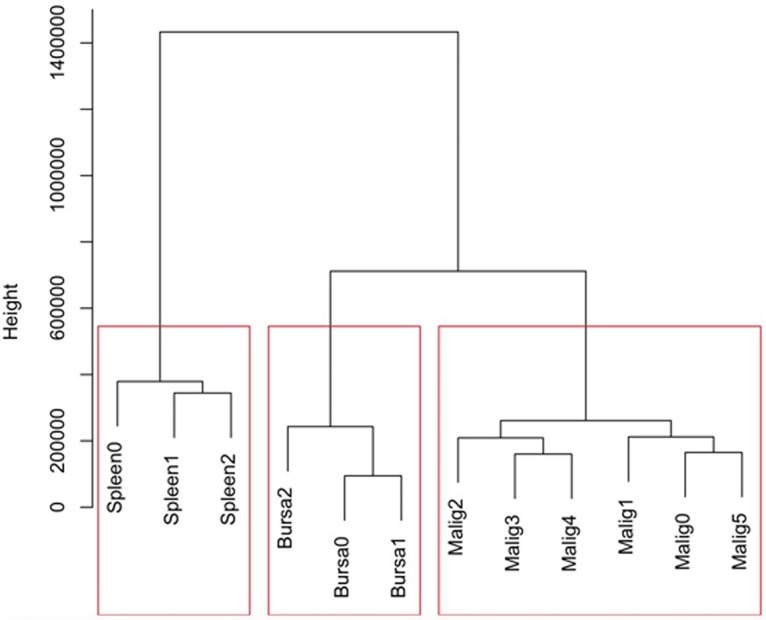
Ward hierarchical clustering by Euclidean distance between the malignant bursa samples (Malig-0 to Malig-5) and the control groups of nonmalignant bursa and splenic B-cell samples (Bursa-0 to Bursa-2 and Spleen-0 to Spleen-2). The result graphically showed that the malignant group was relatively more comparable to the nonmalignant bursa group, in contrast to the splenic B-cell group.

Next-generation sequencing (NGS) read files of the individual RNA samples for the LL-like lymphomas, the control groups of normal bursal tissues, and splenic B cells were deposited in the NCBI SRA database under assigned project accession number PRJNA543277 (https://www.ncbi.nlm.nih.gov/sra/ PRJNA543277). The read data postnormalization are shown in Fig. 2, illustrating the read distributions and variability among the samples. No obvious difference was observable between the lymphoma group (Malig-0 to Malig-5) and either of the control groups (Spleen-0 to Spleen-2 and Bursa-0 to Bursa-2) in distribution, variability, and median values. However, Ward hierarchical clustering analysis was further conducted using Euclidean distance to generate a distance matrix. New clustering analyses showed low biological variability among samples within each of the three biological sample groups, but the splenic B cell group was clearly more distant from the malignant and nonmalignant bursa groups (Fig. 3). Thus, the subsequent comparisons were made between the malignant and nonmalignant bursa tissue groups only, except for the overall gene expression profiling of the LL-like lymphomas and the two control groups of normal bursa tissues and splenic B cells.

### Gene expression profile of the LL-like lymphoma samples.

**FIG 4:**
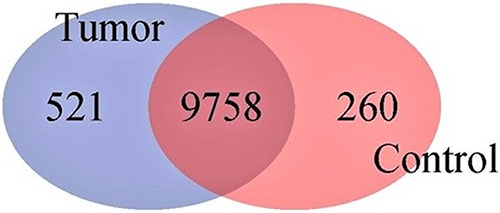
Venn diagram graphically illustrating profile statistics of genes identified by RNA-Seq between the spontaneous lymphoid lymphoma samples and the combined control samples (bursa and splenic B cells). Although most of the genes were expressed in both groups, hundreds of genes were exclusively expressed either in the tumor (lymphoid leukosis-like lymphomas) or the normal control samples.

A total of 10,539 genes/transcripts were identified with a minimum pass filter read count of 10 or more in at least four of the twelve sequenced samples simultaneously. Of those, 521 and 260 expressed genes were exclusively observed in the LL-like lymphoma (tumor) samples and the normal control samples (both normal bursa tissues and splenic B cells), respectively (Fig. 4).

### Identification of differentially expressed genes between the LL-like lymphoma tissues and the normal control of nonmalignant bursa tissues.

The differential expression analysis was conducted between the malignant (LL-like lymphomas) and the nonmalignant bursa groups due to the relatively close compatibility between the LL-like lymphomas and this nonmalignant bursa control group (Fig. 3). A total of 923 genes/transcripts was identified differentially expressed between the LL-like lymphomas and normal bursa control group (0.5 < log_2_ fold change < −0.5 and *P* < 0.001). Of those, 196 genes were significantly more highly expressed and 727 genes were significantly less expressed in the LL-like lymphomas tissues than those in the control group of normal bursa tissues (see Table S1 in the supplemental material). A subset of the top genes significantly upregulated and downregulated was further closely examined and classified into categories of known functions, which include oncogenes, tumor suppressor genes, virus-associated genes, and immune response and cytokine genes. The oncogene category includes upregulated T-cell lymphoma invasion and metastasis 2 (*TIAM2*) and B-cell CLL/lymphoma 9-like (*BCL9L*) genes along with 11 other up- or downregulated oncogenes; the key tumor suppressor genes include downregulated tumor protein p63 (*TP63*) and suppression of tumorigenicity 14 (*ST14*) genes, along with 4 other genes. The key immune response cytokine genes include upregulated interleukin-18 (*IL-18*), class I histocompatibility antigen, F10 alpha chain-like (*HA1F*), and CD247 molecule (*CD247*), along with 15 other up- or downregulated genes (Table 3).

**TABLE 3:** Selected differentially expressed genes with high statistical significance and known functions between the LL-like lymphomas and the control group of normal bursa tissues

Ensemble ID^*a*^	Gene name	Gene description	Log_2_ fold change (M/N)^*b*^	*P* value	Fold change direction
Oncogenes					
16391	CNKSR2	Connector enhancer kinase suppressor of Ras 2	2.7	1.67E−06	Up
36005	TIAM2	T-cell lymphoma invasion and metastasis 2	2.5	3.54E−20	Up
27789	RAB37	Ras-related protein Rab-27-like	1.5	1.23E−11	Up
16817	RASA3	RAS p21 protein activator 3	1.3	5.66E−13	Up
14277	RHOH	Ras homolog family member H	1.3	2.37E−07	Up
07685	BCL9L	B-cell CLL/lymphoma 9-like	1.2	6.48E−05	Up
14913	ROS1	ROS proto-oncogene 1, receptor tyrosine kinase	−8.4	4.19E−69	Down
11664	RASSF6	Ras association domain family member 6	−7.9	4.76E−53	Down
06076	RASGEF1C	RasGEF domain family, member 1C	−3.6	2.88E−18	Down
36883	MET	Proto-oncogene, receptor tyrosine kinase	−3.5	2.12E−26	Down
03129	RAB11FIP1	RAB11 family interacting protein 1 (class 1)	−3.5	6.97E−15	Down
00769	RAB7B	RAB7B, member RAS oncogene family	−2.5	9.11E−14	Down
03503	MYBL2	MYB proto-oncogene like 2	−1.0	1.92E−05	Down
Tumor suppressor genes					
11715	HSPA2	Heat shock 70-kDa protein 2	3.6	5.09E−15	Up
17071	HSPH1	Heat shock 105-kDa/110-kDa protein 1	1.2	4.57E−05	Up
32832	DNAJC16	DNAJ heat shock protein family member C16	1.0	4.77E−06	Up
07324	TP63	Tumor protein p63	−2.2	2.02E−06	Down
01331	ST14	Suppression of tumorigenicity 14	−1.7	3.83E−09	Down
03855	SRC	v-src avian sarcoma (Schmidt-Ruppin A-2) viral oncogene homolog	−1.6	6.14E−09	Down
Virus-associated genes					
30025	FABP4	Fatty acid binding protein 4	3.4	4.79E−08	Up
14860	YES1	y-Yes-1 Yamaguchi sarcoma viral oncogene homolog 1	−2.8	1.81E−31	Down
16059	ETS2	v-ets avian erythroblastosis virus E26 oncogene homolog 2	−2.5	4.21E−22	Down
31529	ERBB2	v-erb-b2 avian erythroblastic leukemia viral oncogene homolog 2	−2.1	8.95E−07	Down
38154	YAP1	Yes-associated protein 1	−1.3	2.51E−05	Down
03670	MAFB	v-maf avian musculoaponeurotic fibrosarcoma oncogene homolog B	−1.2	9.92E−09	Down
Immune response genes and cytokines					
14585	ENSGALG14585	Chemokine	2.8	3.20E−17	Up
30270	CD1C	CD1c molecule	2.7	1.02E−15	Up
12545	CYTIP	Cytohesin 1 interacting protein	2.4	5.55E−11	Up
12292	BANK1	B-cell scaffold protein with ankyrin repeats 1	2.0	9.65E−08	Up
07418	CD3D	CD3d molecule, delta	1.9	5.22E−05	Up
07874	IL18	Interleukin-18	1.9	6.25E−06	Up
26466	HA1F	Class I histocompatibility antigen, F10 alpha chain-like	1.6	5.90E−07	Up
15441	CD247	CD247 molecule	1.6	2.27E−05	Up
19322	TNFRSF13C	Tumor necrosis factor receptor superfamily, member 13C	1.4	9.25E−05	Up
28496	NFKBID	NF-kappa-B-inhibitor delta-like	1.2	1.60E−06	Up
09963	LYZ	Lysozyme (renal amyloidosis)	−7.6	2.60E−62	Down
08552	MAL	Mal, T-cell differentiation protein	−7.2	8.52E−53	Down
15348	ALCAM	Activated leukocyte cell adhesion molecule	−5.5	3.02E−62	Down
11668	IL8L1	Interleukin 8-like 1	−4.0	6.37E−18	Down
08554	IL17REL	Interleukin 17 receptor E-like	−4.0	3.89E−12	Down
37851	KK34	Interleukin-like	−3.6	4.81E−09	Down
26663	CX3CL1	Chemokine (C-X3-C motif) ligand 1	−3.3	1.09E−12	Down
06346	CXCL14	C-X-C motif chemokine ligand 14	−3.2	2.59E−07	Down
38000	CX3CR1	Chemokine (C-X3-C motif) receptor 1	−3.2	3.96E−12	Down
01405	IRF6	Interferon regulatory factor 6	−3.1	3.98E−08	Down
11418	CCR6	C-C motif chemokine receptor 6	−3.0	5.32E−07	Down
21627	IFI27L2	Interferon, alpha-inducible protein 27-like 2	−3.0	5.71E−15	Down
09392	TLR5	Toll-like receptor 5	−2.8	4.70E−19	Down
07174	TNFSF15	Tumor necrosis factor superfamily member 15	−2.8	3.31E−09	Down
29940	IL-1beta	Interleukin-1β	−2.6	1.03E−08	Down
28466	IL34	Interleukin-34	−2.6	1.77E−08	Down
25599	CD24	CD24 molecule	−2.6	7.91E−05	Down
43044	IL1R1	Interleukin-1 receptor, type 1	−2.6	9.73E−19	Down
16785	IL1RL1	Interleukin-1 receptor-like 1	−2.4	1.70E−07	Down
26098	IL8L2	Interleukin-8-like 2	−2.4	1.89E−05	Down
05305	ACKR2	Atypical chemokine receptor 2	−2.3	3.30E−12	Down
03733	LIFR	Leukemia inhibitory factor receptor alpha	−2.2	6.10E−06	Down
11295	SOCS2	Suppressor of cytokine signaling 2	−2.1	6.21E−06	Down
37413	IL7	Interleukin-7	−1.9	1.40E−05	Down
00884	CXXC5	CXXC finger protein 5	−1.9	1.22E−08	Down
17119	TNFSF19	Tumor necrosis factor receptor superfamily member 19	−1.8	3.95E−06	Down
09612	TGFB2	Transforming growth factor, beta 2	−1.7	5.49E−10	Down
03136	IKZF2	IKAROS family zinc finger 2	−1.7	9.74E−05	Down
41621	LY6E	Lymphocyte antigen 6 complex, locus E	−1.6	3.39E−09	Down
09179	TNSF10	Tumor necrosis factor superfamily member 10	−1.5	3.23E−05	Down
11446	TNFAIP2	TNF-α-induced protein 2	−1.5	1.50E−05	Down
06407	TNFRSF23	Death domain-containing tumor necrosis factor receptor superfamily member 23	−1.5	3.42E−05	Down
30005	IGSF1	Immunoglobulin superfamily, member 1	−1.2	5.69E−06	Down

a Gene identifiers (ID) are truncated forms of their designations in the ENSEMBL database, e.g., 16391 is ENSGALG0000016391, 36005 is ENSGALG0000036005, etc.

b M refers to malignant group, that is, the LL-like lymphoma group; N stands for nonmalignant group, that is, the control group of normal bursa tissues.

### GO term and pathway enrichment by lists of differentially expressed genes between the LL-like tumors and the normal bursal controls.

**FIG 5:**
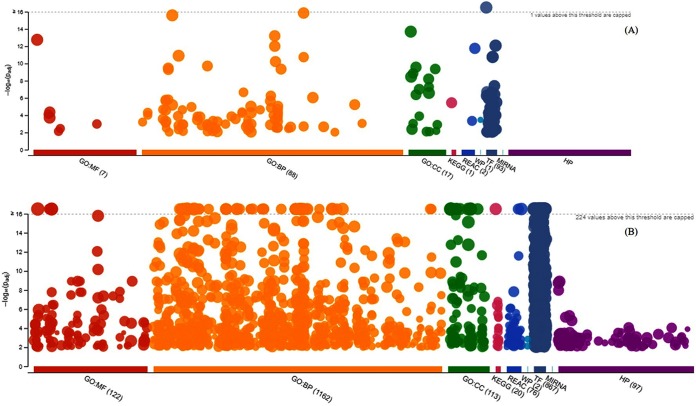
Manhattan plot illustrating the differentially expressed gene-enriched GO terms (MF, molecular function; BP, biological process; and CC, cellular component) and KEGG pathways across reactome pathways (REAC), WiKi-Pathways (WP), transcription factor (TF), microRNA target base (MIRNA), and human phenotype ontology (HP) term categories. (A) Upregulated genes enriched in GO terms and pathways. (B) Downregulated genes enriched in GO terms and pathways. −log_10_(Padj), enrichment score calculated using the formula −log_10_(false discovery rate).

The upregulated genes were enriched in more than 100 Gene Ontology (GO) terms and a few pathways, while the downregulated genes were enriched in more than 1,000 GO terms and more than 100 pathways (Fig. 5). Both lists of differentially expressed genes were enriched across key molecular function, biological process, and cellular component terms (a complete list of GO terms and pathway enrichment outputs for the upregulated and downregulated genes is given in Table S2). Some of the identified most differentially expressed genes of known functions are involved in key GO terms and pathways, which include signal transduction (top oncogene category), apoptotic signaling pathway and regulation of apoptotic signaling pathway (tumor suppressor category), regulation of RNA biosynthetic process and regulation of nucleic acid-templated transcription (viral associated genes), and Toll-like receptor signaling pathways (immune response and cytokine gene category). A complete list of GO terms and pathways that are involved with some of the most differentially expressed genes is detailed in Table 4.

**TABLE 4:** GO terms and pathways involved with some of the top differentially expressed genes between the LL-like lymphomas and normal control group of nonmalignant bursa tissues

Domain^*a*^	GO term	Gene(s)	Up^*b*^	Genes	Down^*c*^
Oncogenes					
BP	Signaling	BCL9L, RHOH, CNKSR2, RASA3	4	RAB7B, RASGEF1C, RASSF6, ROS1, MET	5
BP	Biological regulation	BCL9L, RHOH, CNKSR2, RASA3, TIAM2	5	RAB7B, RASGEF1C, RASSF6, ROS1, MET, RAB11FIP3	6
BP	Regulation of biological process	BCL9L, RHOH, CNKSR2, RASA3, TIAM2	5	RAB7B, RASGEF1C, RASSF6, ROS1, MET, RAB11FIP3	6
BP	Regulation of cellular process	BCL9L, RHOH, CNKSR2, RASA3, TIAM2	5	RAB7B, RASGEF1C, RASSF6, ROS1, MET, RAB11FIP3	6
BP	Signal transduction	BCL9L, RHOH, CNKSR2, RASA3	4	RAB7B, RASGEF1C, RASSF6, ROS1, MET	5
Tumor suppressor genes					
BP	Intracellular estrogen receptor signaling pathway		0	SRC, TP63	2
BP	Apoptotic signaling pathway	HSPH1	1	SRC, TP63	2
BP	Regulation of apoptotic signaling pathway	HSPH1	1	SRC, TP63	2
Virus-associated genes					
BP	Regulation of gene expression		0	MAFB, YES1, ETS2, ERBB2, YAP1	5
BP	Nucleic acid-templated transcription		0	MAFB, YES1, ETS2, ERBB2, YAP1	5
BP	Transcription, DNA templated		0	MAFB, YES1, ETS2, ERBB2, YAP1	5
BP	Regulation of RNA biosynthetic process		0	MAFB, YES1, ETS2, ERBB2, YAP1	5
BP	Regulation of nucleic acid-templated transcription		0	MAFB, YES1, ETS2, ERBB2, YAP1	5
BP	Regulation of transcription, DNA templated		0	MAFB, YES1, ETS2, ERBB2, YAP1	5
BP	Positive regulation of transcription, DNA templated		0	YES1, ETS2, ERBB2, YAP1	4
BP	Positive regulation of gene expression		0	YES1, ETS2, ERBB2, YAP1	4
BP	Positive regulation of RNA biosynthetic process		0	YES1, ETS2, ERBB2, YAP1	4
BP	Positive regulation of nucleic acid-templated transcription		0	YES1, ETS2, ERBB2, YAP1	4
Immune response and cytokine genes					
BP	Regulation of signaling receptor activity	IL18	1	TNFSF15, TGFB2, IL8L1, IL8L2, IL34, IL-1BETA	6
BP	Cytokine-mediated signaling pathway	IL18	1	LIFR, ACKR2, IL17REL, CCR6, IL8L1, IL1RL1, IL8L2, IL1R1	8
BP	Cell activation	IL18, BANK1	2	CCR6, IL8L1, IL1RL1, IL8L2, IL-1BETA	5
BP	Leukocyte activation	IL18, BANK1	2	CCR6, IL8L1, IL1RL1, IL8L2, IL-1BETA	5
BP	Regulation of signaling	IL18, BANK1	2	CXXC5, TNFSF15, TLR5, TGFB2, SOCS2, IL8L1, IL1RL1, IL8L2, IL34, IL-1BETA	10
BP	Regulation of signal transduction	IL18, BANK1	2	CXXC5, TNFSF15, TLR5, TGFB2, SOCS2, IL8L1, IL1RL1, IL8L2, IL34, IL-1BETA	10
BP	Immune system process	IL18, BANK1, CD247	3	CXCL14, TNFSF15, TLR5, TGFB2, CCR6, IL8L1, ALCAM, IL1RL1, IL8L2, CX3CL1, IL34, IL-1BETA	12
BP	Regulation of immune system process	IL18, BANK1, CD247	3	CXCL14, TLR5, TGFB2, CCR6, IL8L1, IL1RL1, IL8L2, IL34, IL-1BETA	9
MF	Signaling receptor binding	IL18, BANK1, CD247	3	LIFR, CXCL14, TNFSF15, TLR5, TGFB2, SOCS2, IL8L1, IL8L2, CX3CL1, IL34, IL-1BETA	11
BP	Signaling	IL18, BANK1, CD247, CD3D	4	CXXC5, LIFR, ACKR2, CXCL14, TNFSF15, IL17REL, TLR5, TGFB2, SOCS2, CCR6, IL8L1, IL1RL1, IL8L2, IL34, IL-1BETA, IL1R1	16
BP	Signal transduction	IL18, BANK1, CD247, CD3D	4	CXXC5, LIFR, ACKR2, TNFSF15, IL17REL, TLR5, TGFB2, SOCS2, CCR6, IL8L1, IL1RL1, IL8L2, IL34, IL-1BETA, IL1R1	15
BP	Immune response	IL18, CD247	2	CXCL14, TNFSF15, TLR5, TGFB2, CCR6, IL8L1, ALCAM, IL1RL1, IL8L2, CX3CL1, IL-1BETA	11
BP	Positive regulation of immune system process	IL18, CD247	2	CXCL14, TLR5, IL8L1, IL1RL1, IL8L2, IL34, IL-1BETA	7
BP	Cell surface receptor signaling pathway	IL18, CD247, CD3D	3	LIFR, ACKR2, IL17REL, TGFB2, SOCS2, CCR6, IL8L1, IL1RL1, IL8L2, IL1R1	10
keg	KEGG pathways	IL18, CD1C, TNFRSF13C	3	LIFR, CXCL14, TNFSF15, TLR5, TGFB2, CCR6, IL8L1, ALCAM, IL8L2, CX3CL1, IL-1BETA, IL7, CX3CR1, IL1R1	14
keg	Cytokine-cytokine receptor interaction	IL18, TNFRSF13C	2	LIFR, CXCL14, TNFSF15, TGFB2, CCR6, IL8L1, IL8L2, CX3CL1, IL-1BETA, IL7, CX3CR1, IL1R1	12
BP	Chemokine-mediated signaling pathway		0	ACKR2, CCR6, IL8L1, IL8L2	4
BP	Leukocyte migration		0	CXCL14, CCR6, IL8L1, IL8L2, IL-1BETA	5
BP	Regulation of leukocyte migration		0	CXCL14, CCR6, IL8L1, IL8L2	4
keg	Toll-like receptor signaling pathway		0	TLR5, IL8L1, IL8L2, IL-1BETA	4

a MF, molecular function; BP, biological process; keg, KEGG pathways.

b Up, upregulated.

c Down, downregulated.

## DISCUSSION

Spontaneous LL-like lymphomas were reported 4 decades ago, having been observed in experimental lines of chickens in the absence of exogenous ALV but with the presence of endogenous ALV (6, 7). Subsequent studies reported that Marek’s disease virus serotype 2, commonly used as one of the bivalent or trivalent Marek’s disease vaccines, augments the incidence of lymphoid leukosis induced by exogenous ALV (41) and the incidence of LL-like lymphomas in chickens free of both exogenous and endogenous ALVs (11, 43).

The putative mechanism had been postulated by researchers encompassing multiple possibilities, whereby MDV-2 could exert influence on bursal cells, which might subsequently modulate the transformation process of B cells alone or in combination with the endogenous, exogenous, or both ALVs. In fact, integration of exogenous herpesviruses into the host genome activates expression of endogenous retroviral genes within the host, as evidenced by Epstein-Barr virus (EBV) (53–55) and Marek’s disease virus (56). Reportedly, MDV-2 only infects and persists in ALV-transformed B cells (45). This supports a model of intracellular cooperation between MDV and ALV resulting in the augmentation of lymphoma development (45). Typically, ALV-E alone demonstrates little to no oncogenicity (24), possibly due to weak promoter activity of the LTR. Coinfection of MDV-2 with ALV results in activation of the retrovirus long terminal repeat by MDV-2. Studies have shown that MDV-2 activates the Rous sarcoma virus LTR (RSV-LTR) promoter 2- to 5-fold more efficiently than serotype 1 MDV (MDV-1) or serotype 3 MDV (MDV-3) (57). Similarly, MDV activates the ALV-LTR promoter, leading to increased expression of ALV RNA, proteins, and infectious viruses in cultured cells (44).

The ALV LTR serves as either a promoter or activator of c-*myc* (58, 59), c-*erbB* (60), and c-*myb* when infection occurs at embryonation (61). The Myc transcription factor induces cell growth and proliferation by influencing the expression of cell cycle regulatory genes (62). ALV induces bursa lymphomas in lymphoma-susceptible strains of chickens after proviral integration within the c-*myc* gene and subsequent expansion of Myc-overexpressing lymphocytes within transformed follicles (58, 63). Transformed follicles grow much more rapidly than normal follicles following ALV infection. This is not solely due to overexpression of Myc; however, susceptible transformed follicles grow much larger than normal ones because following ALV infection, B-cell differentiation and bursal emigration are blocked at an embryonic stage (64). It is speculated that endogenous ALV LTR genes integrate near c-*myc* and activate c-*myc* expression similarly to exogenous ALV LTR. The c-*myc* transcription levels, however, may be lower following endogenous ALV-LTR integration than exogenous ALV-LTR (65).

This report, in part, characterized the two endogenous ALV field isolates, designated AF227 and AF229, which were isolated from commercial broiler breeder flocks after the spontaneous LL-like lymphomas were observed on the farm. To verify if the new ALV-E isolates alone or in combination with the MDV-2 vaccine are responsible, to an extent, for the incidence of spontaneous LL-like lymphomas, birds from different commercial lines, other than the flocks from which AF227 and AF229 were isolated (those flocks of birds were no longer available at the time of the challenge trials of this study), and a fully susceptible chicken line, developed and maintained on the ADOL farm and known as the RFS line and free of any endogenous *ev* genes (46), were infected with each of the ALV-E field isolates or inoculated with the MDV-2 vaccine or a combination of the ALV-E isolate and the MDV-2 vaccine. There was no incidence of spontaneous LL-like lymphomas observed in the commercial broiler breeder chickens following infection with either AF229 or AF229 with MDV-2 vaccination in this study (Table 2). No significant difference in LL-like lymphoma incidence was detected between the AF227 isolate group and the MDV-2 vaccination group (*P* > 0.05) in the RFS birds, as shown in Table 1. However, each ALV-E isolate in combination with MDV-2 vaccine significantly accelerated the incidences of spontaneous LL-like lymphomas in the RFS birds, in contrast to the sole AF227 isolate infection and the sole MDV-2 vaccination groups (Tables 1 and 2; *P* < 0.05).

Genetic resistance to ALV is multifactorial and depends upon resistance to infection and resistance to tumor development (32, 33, 39). The commercial broiler breeder chickens appear to be resistant to tumor development since they were susceptible to ALV-E infection, as evidenced by the presence of the ALV-E receptor TVB*S1/*S1, and they were viremia tolerant following infection at embryonation. Parghi et al. (66) identified that in ALV-resistant chickens, ALV LTR-enhanced c-*myc* gene expression is reduced, resulting in normal B-cell differentiation, normal follicle development, and posthatch bursal emigration in resistant transformed bursa follicles. We did not analyze bursa tissue from the commercial broiler breeder chickens to determine the level of gene transcription in transformed bursa follicles. It would, however, be interesting to note the levels of gene expression in transformed bursa follicles between susceptible and resistant chickens.

We did assess the whole-genome transcriptomic levels between the LL-like bursa lymphomas collected from the RFS birds after SB-1 and AF227 inoculation and the normal control group of bursa tissue samples of the same line of birds. Next-generation sequence analysis resulted in hundreds of differentially expressed genes/transcripts for the first time between LL-like lymphoma samples and the normal bursa tissue samples, which include genes of known function in the categories of oncogenes, tumor suppressor genes, virus-associated genes, and immune response and cytokine genes. A minimum of six oncogenes were upregulated and seven oncogenes downregulated in the spontaneous LL-like lymphoma samples, in contrast to the normal control group of nonmalignant bursa tissues and in addition to up- and downregulated genes with categorically known functions of tumor suppression, viral functions, immune responses, and activities of cytokines (Table 3). Further bioinformatics analyses showed that some of those differentiated genes reportedly are involved in key GO terms and pathways, including signaling pathways, signal transduction, immune response, and KEGG pathways.

The upregulated known oncogenes and downregulated tumor suppressor genes represented only a small subset of the 923 genes significantly expressed between the LL-like lymphoma samples and the nonmalignant normal bursa tissues. Sequence analyses of higher depth than those of this study and additional LL-like lymphoma samples for such analyses are warranted and necessary to advance the understanding of the mechanism at genomic levels on what constitutes the LL-like lymphoma susceptibility and how a nonpathogenic subgroup of ALV in conjunction with little or no pathogenic MDV-2 jointly boost the incidence of LL-like lymphomas in susceptible chickens.

In summary, we have isolated two endogenous ALV field isolates capable of inducing spontaneous LL-like lymphomas in susceptible chickens in conjunction with MDV-2. We have characterized the two ALV field isolates at the genomic level, designated the isolates AF227 and AF229. The genomic sequence analyses of the AF227 and AF229 isolates showed these two ALV isolates belong to subgroup E ALV. RNA sequencing analyses of the spontaneous LL-like lymphomas induced by AF227 and SB-1 in an experimental line of birds under controlled conditions and normal bursa tissues of the same genetic line of birds resulted in a total of 923 differentially expressed genes between the two groups. Some of the differentially expressed genes with known functions of oncogenicity and tumor suppression, association with viral functions, immune responses, and cytokine activities are involved with multiple key pathways, including signaling, signal transduction, and KEGG pathways.

## MATERIALS AND METHODS

### Virus isolation.

Plasma, spleen, and liver samples of eight breeders of a broiler breeder flock were received from a commercial farm at which spontaneous lymphoid leukosis-like bursal lymphoma incidences were observed. The samples were tested for virus growth using chicken embryo fibroblasts (CEF) from ADOL-specific pathogen-free lines of chickens, RFS (C/0) and line 0 (C/E), and followed by PCR using viral subgroup-specific primers. Aliquots of plasma, spleen, and liver homogenates were seeded onto secondary CEF from line 0 (9) and line RFS (46) for virus isolation. CEFs were maintained in Leibovitz L-15 medium plus McCoy 5A medium (1:1), supplemented with 1% bovine serum and antibiotics for 10 to 14 days before harvest of cell-free viruses. Two isolates, designated AF227 and AF229, were obtained and were further characterized molecularly and biologically. Total viral DNA from CEF-infected cells was extracted using standard proteinase K, phenol-chloroform extraction procedures. The viral DNA was analyzed by PCR using ALV subgroup-specific primers as described by Silva et al. (67) to determine which subgroup or subgroups of ALV were present in the viral DNA sample.

### Viral DNA sequence analysis.

The proviral DNA samples from the infected CEFs were sequenced at the Research Technology Support Facility, Michigan State University (East Lansing, MI). Contigs were constructed using Sequencer (Gene Codes Corp., Ann Arbor, MI). DNA sequences were aligned using the Clustal W model in the MegAlign program of Lasergene (version 11; DNASTAR, Inc., Madison, WI). Phylogenetic relatedness was also calculated using the MegAlign program. The sequence analyses included the isolates AF227 and AF229, along with the prototype ALV-E virus RAV-0 (24), partial sequence of the recombinant ALV-E strain RAV-60 (68), and ALV subgroup A strain RAV-1 (69).

### Lines of chickens used in the challenge trials.

Chickens from the line RFS were used in this study, since they are free of endogenous viruses, TVB*S1/*S1 homozygous, and thus are fully susceptible to infection of all subgroups of ALV viruses, including the ALV-E subgroup (46). Fertile eggs were also obtained from a commercial broiler breeder, which were incubated on the ADOL farm, and the chickens hatched from the eggs were included in one of the experiments. We note that the fertile eggs obtained this time were from the same commercial broiler breeder from which the specimens were received earlier for the virus isolations of AF227 and AF229, but the fertile eggs received this time for the experiment were from a different broiler flock housed on a different farm rather than the original flock and farm from which the spontaneous LL-like tumor incidences were observed or AF227 and AF229 were isolated. Two challenge trials were conducted. One was with chickens only from the ADOL line RFS and the other with chickens from both ADOL line RFS and the commercial broiler breeder eggs hatched on the ADOL farm. All of the birds in each experiment from each line were housed in a biosafety level 2 facility on the ADOL farm. Feed and water were supplied *ad libitum*.

### A challenge trial to test the pathogenicity of ALV isolate AF227 in RFS chickens.

Sampled fertile eggs from the ADOL line RFS chickens were divided into four groups. One group of the embryos, a control group, was inoculated with 100 μl of sterile PBS, and two of the groups were inoculated with 100 μl of ALV-E isolate AF227 at 1,000 50% tissue culture infectious doses/0.1 ml per bird via yolk sac at 7 DOE. At 1 day of age, the other group of chickens that did not receive any treatment and one of the groups that had received AF227 inoculation at 7 DOE was given 500 PFU each of the MDV-2 vaccine SB-1 intraperitoneally. Chickens of the different treatment groups were housed in separate isolators and monitored up to 50 weeks of age. At 2 weeks of age, blood samples were collected from a subset of chickens from each group, and buffy coats were analyzed for the presence of MDV-2-specific plaques as described by Aly et al. (40).

### A challenge trial to test the pathogenicity of AF227 and AF229 ALV isolates in commercial breeder broilers and RFS chickens.

The received commercial broiler fertile eggs were divided into five groups in incubation. One group received PBS (control), two groups received AF229, and one group received AF227 at 7 DOE. At 1 day of age, the group of birds pretreated with AF227 and the group of birds that did not receive any treatment at 7 DOE, along with one group of birds pretreated with AF229, were vaccinated with MDV-2 as described above. Fertilized eggs from line RFS were divided into three groups. One group was given PBS (control), and the other two groups were given AF227 or AF229 at 7 DOE. The latter two groups were inoculated with SB-1 vaccine at 1 day of age as described above. All chickens were bled at 4 weeks of age, and all surviving chickens were bled at 52 weeks of age prior to termination. The procedures for handling and sampling of the chickens were preapproved by the ADOL Animal Care and Use Committee (ACUC). The experimental chickens were monitored daily throughout the 52-week experiment period, and all moribund chickens were euthanized humanely by following American Veterinary Medical Association-approved methods.

### Virus and antibody assays.

The plasma samples were assayed for infectious exogenous and endogenous ALV as well as the presence of antibody by preestablished procedures described previously by Fadly and Witter (70). The presence or absence of MDV-2 in individual chickens was determined by cocultivation of 1 × 10^6^ buffy coat cells from centrifuged and heparinized blood samples onto duck embryo fibroblasts (DEF), as described previously by Aly et al. (40).

### Pathology examinations.

All chickens that died during experiments or were euthanized at the end of the experiments were subjected to individual necropsy. Lymphomas were diagnosed on the basis of visual and histological examinations of tissues with gross tumors or suspicious microtumors, respectively (3).

### *TVB* genotyping by pyrosequencing.

*TVB* genotypes of all the commercial broilers included in this experiment were determined by pyrosequencing analysis by following procedures described by Zhang et al. (22). Briefly, short PCR amplicons were generated from the broiler DNA samples with a pair of primers, of which one of the primers was biotinylated at the 5′ end. The PCR products then were subjected to binding, shaking, annealing, washing, and denaturation processes. The end products of the PCR amplicons were biotin-labeled and single-stranded DNA, which were then analyzed on a PSQ 96MA pyrosequencer system (Qiagen, Inc., MD) for *TVB* genotypes. There are six commonly observed *TVB* genotypes in commercial birds, which are TVB*S1/*S1, TVB*S1/*S3, TVB*S1/*R, TVB*S3/*S3, TVB*S3/*R, and TVB*R/*R.

### Tissue samples, B-cell isolation, and total RNA extraction.

LL-like bursal lymphoma tissues were collected from six RFS chickens treated with AF227 at 7 DOE and SB-1 on the day of hatch during postmortem examination between 32 and 43 weeks of age (see Table S3 in the supplemental material). Fresh normal bursal tissues from three 3-week-old noninoculated RFS line chickens were also collected. Splenic B cells from three noninfected and age-matched RFS line chickens were isolated using a magnetically activated cell sorting (MACS) cell separation system by following the manufacturer’s instructions (Miltenyi Biotech, San Diego, CA). Briefly, the spleen tissues were first individually homogenized. Separated cells were stained with a fluorescein isothiocyanate (FITC)-conjugated primary antibody. Subsequently, the cells were magnetically labeled with anti-FITC microbeads. The cell suspension was loaded onto a MACS column, and labeled cells were separated via a MACS separator. Total RNA was extracted from tissue homogenates and the B cells using an RNeasy kit as recommended by the manufacturer (Qiagen, Valencia, CA). The last two groups of samples, the normal bursal tissues and the splenic B cells, served as the normal control groups of the LL-like bursal lymphoma samples in RNA-Seq analysis.

### Next-generation sequencing analysis.

Total RNAs of the bursal lymphoma tissue, the normal bursal tissues, and the splenic B cells were subjected to NGS analysis. NGS libraries were built using Illumina’s TruSeq stranded mRNA library preparation kit by following the manufacturer’s instructions (Illumina, San Diego, CA). Sequencing was performed on an Illumina HiSeq 2500 machine running in high-output mode in a 2× 100-bp paired-end format using an Illumina TruSeq PE cluster kit (v3) and TruSeq SBS kit (v3). The raw reads were quality assessed using FastQC, version 0.11.2 (http://www.bioinformatics.babraham.ac.uk/), and adaptors were removed using Trimmomatic, version 0.30 (71). Low-quality bases were trimmed using custom Python scripts to remove the first 13 nucleotides, and Sickle v1.33 (72) was used, under a sliding window with an average quality score of 30, for removal of reads with N nucleotides and reads that were under the 50-bp minimum read length threshold. The good-quality reads were mapped to a combined reference sequence containing the chicken (galGal4) genome (73), SB-1 (GenBank accession no. HQ840738.1) (74), and Rous sarcoma virus (NCBI reference sequence NC_001407.1) (75) with Ensembl annotation (76) using TopHat2, version 2.0.8b (77), and Bowtie2, version 2.1.0 (78). This resulted in approximately 33,806,146 minimum reads, 55,558,773 median reads, and 118,577,764 maximum reads in the alignments of the 12 sample libraries. The alignments were subjected to subsequent analysis with CuffDiff2, version 2.2.0 (79), to identify genes that were differentially expressed between the LL-like bursal lymphoma group and the control group. Ensembl gene identifiers for the differentially expressed genes were used to form the upregulated and downregulated gene lists, which were used as the input for Gene Ontology and pathway enrichment analyses using both custom R scripts and the g:Profiler online resources (80).

### Reconstruction of AF227 genome from the LL-like lymphoma RNA-Seq data.

The RNA-Seq reads of the LL-like lymphoma samples were aligned to the NCBI viral genomes database (81) using BLAST+, version 2.2.28 (82). All reads that matched to an annotated avian virus were retained, pooled, and then assembled into a reconstructed version of the AF227 genome with Trinity, version 20140413p1 (83). All next-generation sequence data processes were performed at the Michigan State University High-Performance Computing Facility (East Lansing, MI).

### Data availability.

The complete genomic sequences have been deposited in GenBank under accession numbers MF817820 for AF227, MF817821 for AF229, MF817822 for RAV-0, MF817823 for a partial RAV-60 sequence, and MF926337 for RAV-1.

## Supplementary Material

Supplemental file 1
